# Optimization of a rapid one-step platelet-rich plasma preparation method using syringe centrifugation with and without carprofen

**DOI:** 10.1186/s12917-020-02350-2

**Published:** 2020-05-06

**Authors:** Jutarat Apakupakul, Panpicha Sattasathuchana, Phongsak Chanloinapha, Naris Thengchaisri

**Affiliations:** 1 Graduate Student in Veterinary Clinical Studies, Faculty of Veterinary Medicine, Kasetsart 462 University, Bangkok, 10900 Thailand; 2 Department of Companion Animal Clinical Sciences, Faculty of Veterinary Medicine, Kasetsart University, Bangkok, 10900 Thailand; 3 Kasetsart University Veterinary Teaching Hospital, Faculty of Veterinary Medicine, Kasetsart University, Bangkok, 10900 Thailand

**Keywords:** Blood, Centrifugation, Dog, Platelet-rich plasma, Carprofen

## Abstract

**Background:**

Carprofen and platelet-rich plasma (PRP) are widely used in small animal clinical practice. Separation layers have been used during blood centrifugation to increase platelet yield. The objectives of this study were to (1) identify the optimal centrifugation force for the one-step PRP preparation, (2) determine whether there is an advantage to using carprofen in one-step PRP preparation, and (3) compare platelet morphology from one-step PRP preparation with and without carprofen. We hypothesized that injectable carprofen (emulsion formula) could be used successfully as the separation layer in PRP preparation.

**Results:**

Samples from 14 healthy dogs were used to determine the optimal centrifugation force using one-step PRP preparation in a disposable syringe without carprofen, with forces set at 300, 500, 700, 900, 1100, 1300, and 1500 xg for 5 min. Optimum centrifugation force, plasma volume, and platelet concentrations of one-step PRP preparation were found and recovered at 900 xg, 1.9 ± 0.28 ml, and 260.50 ± 58.39 X 10^3^ cell/μl, respectively. Samples from 12 healthy dogs were used to determine the optimal force (with forces set at 300, 500, 700, and 900 xg) for 5 min using one-step PRP preparation with carprofen. Optimum centrifugation force, plasma volume, and platelet concentrations for one-step PRP preparation with carprofen were found and recovered at 500 xg, 0.62 ± 0.16 ml and 948.50 ± 261.40 X 10^3^ cell/μl, respectively. One-step PRP preparation with carprofen increased the platelet yield from baseline by 1.76 and 4.95 fold, respectively. Samples from 3 healthy dogs were used to observe platelet morphologies after centrifugation by scanning electron microscopy. Images of platelets on glass slides from both preparation methods revealed pseudopods emerging from the margins of the discoid platelets.

**Conclusions:**

One-step PRP centrifugation both with and without carprofen increased the platelet yield, but using carprofen (emulsion formula) as a separation layer resulted in a higher platelet yield. The clinical usefulness of PRP products from these methods should be further investigated.

## Background

The use of platelet-rich plasma (PRP) in clinical practice has increased recently [1–8]. PRP also is used in orthopedic surgery to treat bone, tendon, and ligament injuries [8–14]. Several clinical studies have found that PRP injections can improve functional outcomes and reduce symptoms when compared with hyaluronic acid and placebo controls [13]. Intra-articular injections of PRP can be useful in extracellular matrix remodeling; have the potential to promote cell proliferation, chemotaxis, cell differentiation, and angiogenesis; and can be a potent source of regenerative growth factors [12, 13, 15–17]. Several growth factors are released by PRP, such as platelet-derived growth factors, epidermal growth factors, fibroblast growth factors, insulin-like growth factors, vascular endothelial growth factors, transforming growth factors, and keratinocyte growth factors [8]. PRP usually is prepared using a one- or two-step centrifugation technique [1–8]. The preparation time for the one-step technique is shorter than for the two-step technique [2]. The one-step technique can be manually completed using a benchtop centrifuge, a single sterile pipette, and a single sterile tube [1]. Although the two-step centrifugation technique can increase platelet concentration [2], it is time consuming and may increase a chance of bacterial contamination. Commercially available PRP kits have been developed for semiclosed preparation in a one-step centrifugation, but they are expensive and require specialized equipment.

Carprofen is a selective cyclooxygenase-2 inhibitor and widely used by veterinarians as an analgesic after orthopedic procedures [18–20]. It has a positive influence on the healing of cartilage [20]. Gradient centrifugations using insoluble emulsion have been used to separate PRP from whole blood [21]. The separation properties of emulsion are due to its phospholipid interface and water interface [22]. In one study, carprofen microspheres were prepared with an emulsion solvent evaporation technique using cellulose acetate phthalate as the polymer dissolved in dichloromethane (1.5% w/v) [23]. Injectable carprofen (emulsion formula) can be applied as a separation layer during PRP preparation, but currently, there is no standardized protocol for optimizing PRP preparation with a separation layer. Therefore, the objectives of this study were to (1) identify the optimal centrifugation force for the one-step PRP preparation, (2) compare the one-step syringe centrifugation technique with and without carprofen used as the separation layer, and (3) compare platelet morphology after one-step PRP preparation with and without carprofen.

## Results

**Fig. 1:**
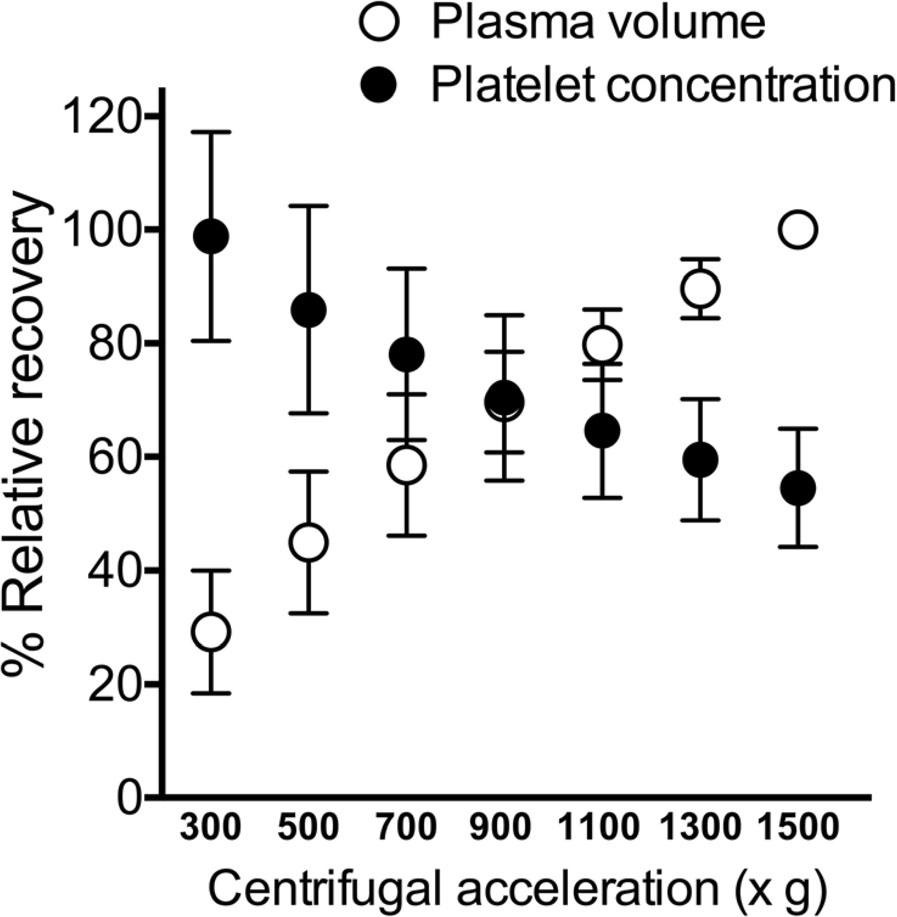
Optimal centrifugation force for one-step PRP preparation. Optimal centrifugation force for one-step PRP preparation was identified at the intercept between % relative recovery of platelet concentration and plasma volume. The optimum centrifugation force was found to be 900 xg

The mean ± standard deviation (SD) platelet concentration of whole blood before centrifugation for the one-step PRP preparation was 230.79 ± 41.48 X 10^3^ cell/μl. The average fold-increase in platelet concentration ranged from 1.14 to 2.09 (Table 1). PRP centrifugation from 300 xg to 1500 xg led to an upward trend of plasma volume from 29.24 to 89.61% but caused a downward trend in platelet concentration from 98.83 to 54.55%. The optimal centrifugal force for syringe centrifugation was found to be 900 xg, at which the plasma volume and platelet concentration were 1.9 ± 0.28 ml and 260.50 ± 58.39 X 10^3^ cell/μl, respectively (Fig. 1, Table 1). The platelet concentration, percentage of recovery, and platelet fold increase for one-step PRP preparation at 900 xg were significantly different between the centrifugal forces at 300, 500, 1300, and 1500 xg (*p* < 0.0001) (Table 1).

**Table 1:** Effects of different centrifugation forces in one-step platelet-rich plasma preparation (*N* = 14)

Force, xg	Platelet concentration, X 10^3^ cell/μl	Plasma, ml	Recovery, %	Platelet fold-increase^a^
300	376.50 ± 73.52***	0.8 ± 0.33***	56.45 ± 7.59***	2.09 ± 0.18***
500	326.43 ± 73.10***	1.1 ± 0.38***	65.09 ± 6.74***	1.81 ± 0.20***
700	284.43 ± 60.54	1.5 ± 0.38***	75.27 ± 6.85**	1.58 ± 0.17*
900	260.50 ± 58.39	1.9 ± 0.28	81.65 ± 4.91	1.44 ± 0.16
1100	236.56 ± 47.04	2.2 ± 0.23*	88.55 ± 4.44***	1.32 ± 0.11*
1300	214.49 ± 42.77***	2.5 ± 0.19***	93.38 ± 3.96***	1.19 ± 0.11***
1500	205.52 ± 41.57***	2.8 ± 0.15***	96.85 ± 2.54***	1.14 ± 0.11***

^a^From the mean platelet concentration of 230.79 ± 41.48 × 10^3^ cell/μl before centrifugation. Statistical analyses were performed using one-way analysis of variance comparing with the optimal centrifugation force of 900 xg; data expressed as mean ± standard deviation. g = gravitational force. *p < 0.05, **p < 0.01, and ***p < 0.001 in post-hoc tests

**Fig. 2:**
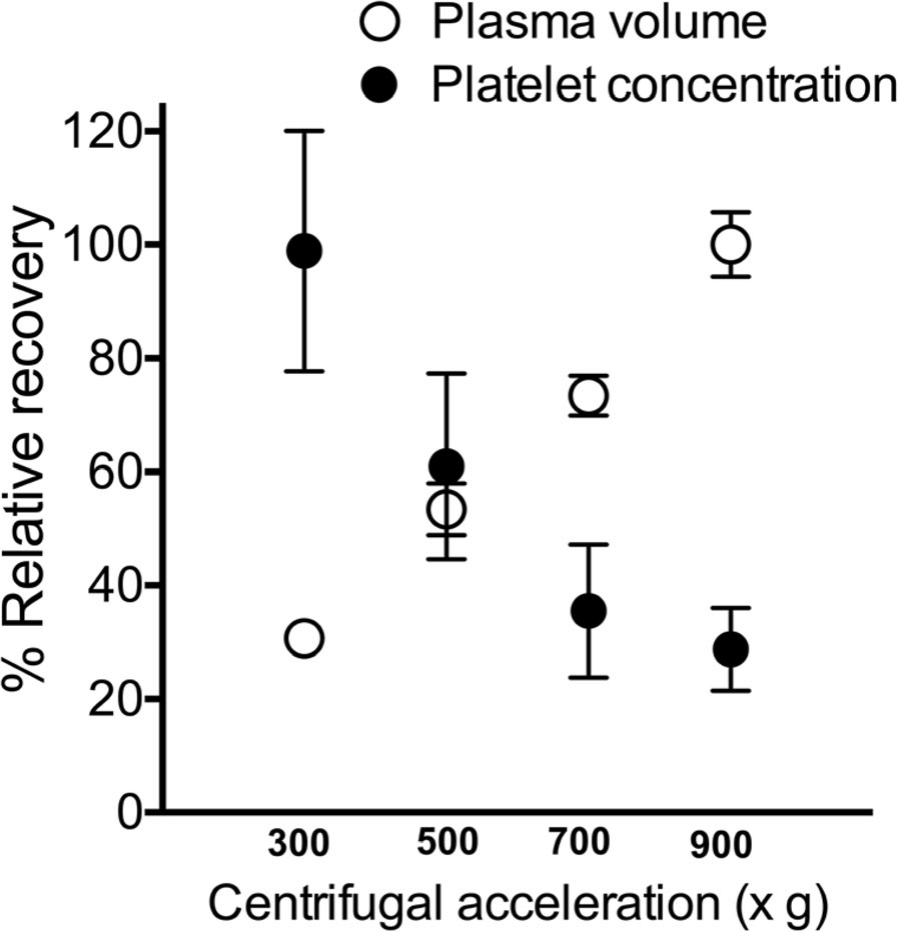
Optimal centrifugation force for one-step PRP preparation with carprofen. Optimal centrifugation force for one-step PRP preparation with carprofen as a separation layer was identified at the intercept between % relative recovery of platelet concentration and plasma volume. The optimum centrifugation force was found to be 500 xg

The mean ± SD platelet concentration of whole blood before centrifugation using the one-step PRP preparation with carprofen was 214.58 ± 53.91 X 10^3^ cell/μl. The average fold-increase in platelet concentration ranged from 2.34 to 8.19 (Table 2). The platelet concentration, platelet fold increase, and percentage of recovery in one-step PRP preparation with carprofen at 500 xg were significantly different from those found at 300, 700, and 900 xg (*p* < 0.0001) (Table 2). The coefficient of variance (CV) in one-step PRP preparation without and with carprofen ranged from 0.82 to 1.71% and 1.87 to 2.42%, respectively (Table 2). As PRP centrifugation was increased from 300 to 900 xg, there was an upward trend in plasma volume, from 30.70 to 92.70%, but a downward trend in platelet concentration, from 98.91 to 28.73% (Fig. 2). The optimal centrifugation force was identified as 500 xg, at which the plasma volume and platelet concentration were 0.62 ± 0.16 ml and 948.50 ± 261.40 X 10^3^ cell/μl, respectively (Fig. 2, Table 3).

**Table 2:** Effects of four different centrifugation forces for one-step platelet-rich plasma preparation with and without carprofen (*N* = 12)

Force, xg	Platelet concentration, X 10^3^ cell/μl	Platelet fold-increase	Recovery, %	Coefficient of variance, %
Without carprofen^a^				
300	391.75 ± 49.55***	2.02 ± 0.28***	56.70 ± 5.22***	0.82
500	328.17 ± 55.36***	1.74 ± 0.16***	69.87 ± 3.84***	1.38
700	290.75 ± 56.69*	1.54 ± 0.17*	83.00 ± 4.70**	1.70
900	260.13 ± 44.85	1.38 ± 0.12	88.66 ± 3.49	1.71
With carprofen^b^				
300	1556.10 ± 339.55***	8.19 ± 0.84***	89.39 ± 7.08***	1.87
500	948.50 ± 261.40	4.95 ± 0.76	97.80 ± 1.72	1.78
700	552.29 ± 187.22***	2.87 ± 0.63***	86.19 ± 2.71***	2.42
900	447.13 ± 116.64***	2.34 ± 0.33***	82.80 ± 3.88***	2.23

^a^Compared with the optimal centrifugation force of 900 xg. ^b^ Compared with the optimal centrifugation force of 500 xg. Platelet concentration, increase and % recovery expressed as mean ± standard deviation. g = gravitational force. *p < 0.05, **p < 0.01, and ***p < 0.001 in post-hoc tests

**Table 3:** Comparison of RBC, WBC, platelet concentration, and plasma volume between both syringe centrifugation methods

Parameters	Whole blood	One-step PRP preparation without carprofen^a^	One-step PRP preparation with carprofen^b^
RBC, X 10^6^ cell/mm^3^	6.15 ± 0.85	1.97 ± 0.37***	0.58 ± 0.24***
WBC, X 10^3^ cell/mm^3^	10.34 ± 2.84	0.36 ± 0.19***	0.72 ± 0.16***
Platelet, X 10^3^ cell/μl	214.58 ± 53.91	260.13 ± 44.85***	948.50 ± 261.40***
Plasma, ml	2.95 ± 0.25	1.97 ± 0.37***	0.62 ± 0.16***

^a^Compared with the optimal centrifugation force of 900 xg. ^b^ Compared with the optimal centrifugation force of 500 xg. Data expressed as mean ± standard deviation. g = gravitational force; PRP = platelet-rich plasma; RBC = red blood cell; WBC = white blood cell. *p < 0.05, **p < 0.01, and ***p < 0.001 in post-hoc tests

**Fig. 3:**
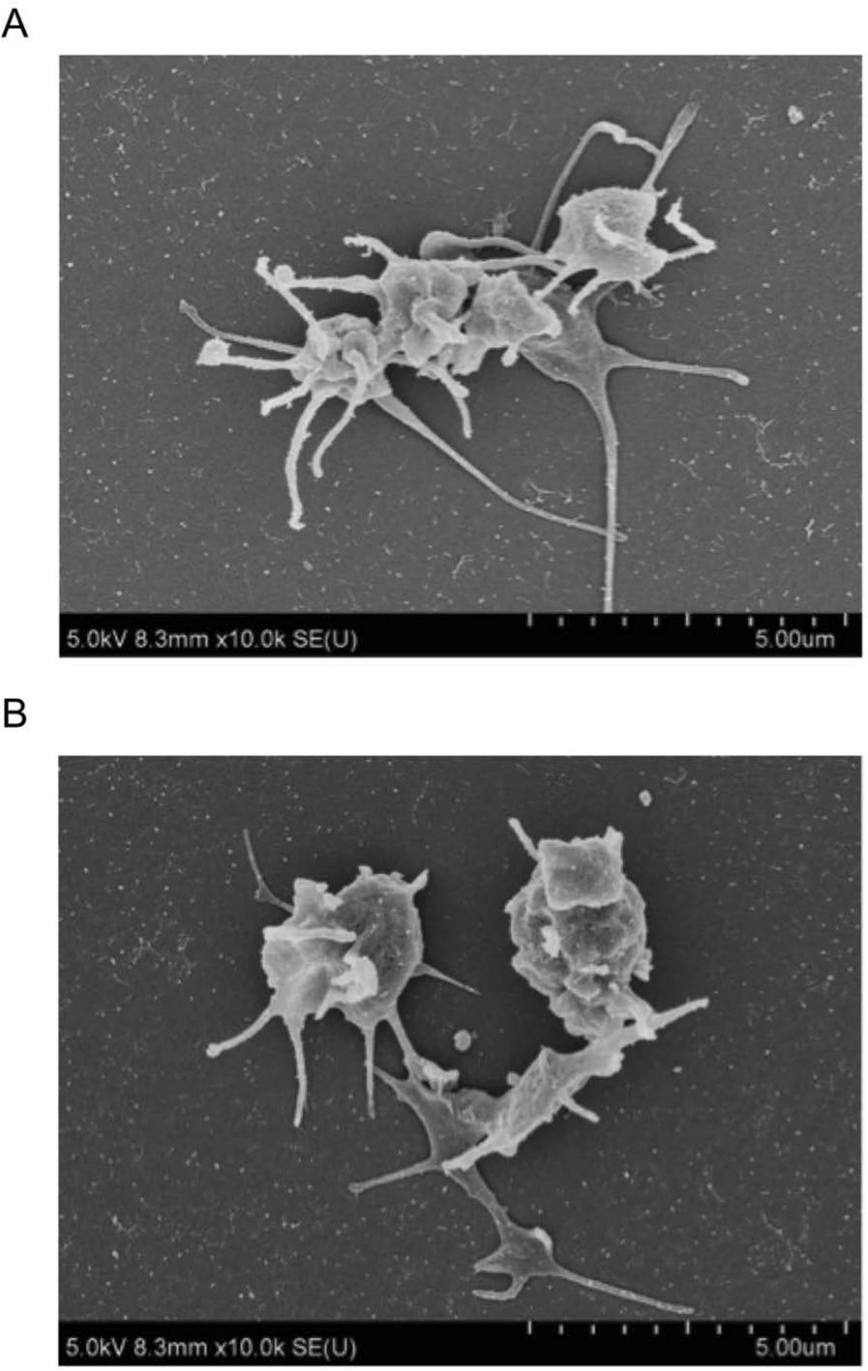
Scanning electron micrographs of platelets. Scanning electron micrographs of platelets in one-step PRP preparation without (**a**) and with (**b**) carprofen. **a** Platelets showed pseudopods emerging from the margins of the discoid platelets (X 10,000). **b** Platelets showed pseudopods emerging from the margins of the discoid platelets (X 10,000)

The mean red blood cell, white blood cell, and platelet concentrations were significantly different between the whole blood and products from PRP without carprofen at 900 xg (*p* < 0.0001) and between whole blood and PRP products with carprofen at 500 xg (*p* < 0.0001; Table 3). The morphology of glass-activated platelets using scanning electron microscopy (SEM) indicated that pseudopods emerging from the margins of the discoid platelets in one-step PRP preparation are similar to that found in one-step PRP preparation with carprofen (Fig. 3).

## Discussion

*Platelet-rich plasma* (PRP) is defined as an autologous biological product derived from whole blood that has been centrifuged to remove red blood cells. PRP is a part of the plasma fraction with a platelet concentration higher than that of whole blood [8]. The efficiencies of red blood cell separation and platelet concentration depend on the density gradient, speed, number of spins, separation time, volume of whole blood, initial platelet concentration, distance between the particles, and the centrifuge rotor to the volume of processed whole blood, including clotting factors [24, 25]. The results of this study show that a one-step PRP preparation using a disposable 6 ml syringe with and without carprofen can separate plasma layers and increase platelet yield. The optimal centrifugal forces with and without carprofen were identified as 500 xg and 900 xg, respectively. The one-step PRP preparation without carprofen led to a 1.38-fold increase in platelet concentration; however, the addition of carprofen led to a 4.95-fold increase. In a previous study, the % CV in one-step PRP preparation was low (< 15%) between sample replicates [26]. In present study, it was confirmed through analysis of % CV that there was comparable precision and variation between the one-step PRP preparation with and that without carprofen [26]. The morphology of platelets indicated that pseudopods were emerging from the margins of the discoid platelets from PRP preparation without carprofen and with carprofen, suggesting normal platelet function.

Centrifugation is regularly used to separate components of a substance on the basis of particle size and density [27–29]. It is applied to separate the blood into cells and plasma supernatant or serum. There are several protocols available to prepare PRP [2, 3, 7, 30–35]. Using leukocyte and fibrin content, PRP can be categorized in to four groups including leukocyte-rich PRP, leukocyte reduced PRP, leukocyte platelet-rich fibrin and pure platelet-rich fibrin [36]. In the present study, a one-step PRP preparation method was used to identify the optimal centrifugation force in a disposable 6 ml syringe. Since there was no special filter for leukocytes used in a single step PRP preparation, our method would produce leukocyte-rich PRP. Moreover, carprofen was applied as a separation layer, dividing plasma and cellular elements. The present method has several advantages, such as increased yield of platelet concentration, reduced red and white blood cell concentrations, and reduced centrifuge force and time.

The efficacy of the one-step [34] and two-step [2] PRP preparation from human blood samples have been previously. A one-step process using 4.5 ml of whole blood with 3.8% trisodium citrate led to a 2.67-fold increase in platelet yield from the baseline value [34]. A two-step process using 4.5 ml of whole blood with 0.5 ml citrate solution led to an increase in platelet concentration by a factor of 5.4–7.3 [37]. In a study of blood samples from dogs, a two-step centrifugation process using 9 ml of whole blood with 1 ml of citrate phosphate dextrose adenine-1 (CPDA-1) obtained a PRP volume of 0.3 ml with a concentration about 5.26–6.94 times higher compared with the original 9 ml whole blood sample In the present study, a one-step centrifugation process using 5 ml of whole blood with 4.5% of CPDA-1 in a disposable syringe led to a 1.76-fold increase in platelet concentration, which was comparable to the results of previous research Interestingly, the one-step process with carprofen led to a 4.95-fold increase in PRP concentration. It should be noted that amount of RBC and WBC contaminations were lower in a one-step PRP preparation with carprofen. Addition of carprofen as a separation layer helped improve plasma separation and increase platelet concentration. Thus, our results demonstrated that higher PRP concentrations could be achieved with only one centrifugation spin.

In the present study, statistic calculator software was applied to compute the appropriate sample size for the one-step PRP preparation to achieve the target power of 90% for clinical study. Nonetheless, different groups of animals were used during the baseline study for the one-step PRP preparation with syringe centrifugation and during the comparison between carprofen-free test and carprofen-based test, leading to the use of several canine blood donors. There are several ways to improve the methodology of the present study that would help minimize the number of canine blood donors enrolled in the study. Studying in any one breed of dogs could lower genetic variations leading to lower variations of the platelet count. The one-step PRP preparation, demonstrated in a present study, requires only small amount of blood (5 mL) for a syringe centrifugation, therefore, it is possible to retrieve a serial blood sampling from the same animal using an indwelling catheter leading to a lower variation between subjects and improving the statistical power of the results. Moreover, the one-step PRP preparation using syringe centrifugation also offers the opportunity to apply an autologous PRP treatment for a small sized dog.

An SEM study revealed that resting platelets had a discoid shape with a smooth surface around 1–3 μm in size [38]. The resting platelets can be activated by shear stress, thrombin, or endothelial membrane-induced morphological changes of platelets [39–42]. Activated platelets become spherical as actin filaments. Cell membranes protrude actin filament known as pseudopods [43]. In the present study, platelet activation was determined using a glass slide. An adhesion and activation of platelet on glass surface was revealed on SEM images demonstrating a discoid shape projecting marginal pseudopods. Pseudopods were identified in the platelets from the PRP preparation using carprofen. Therefore, the use of carprofen as a separation layer had a minimal effect on platelet activation in the present study.

Bacterial contamination is a leading cause of transfusion-related mortality [44]. Conventional methods using two-step PRP preparation increase the risk for environmental bacterial contamination, especially during the transfer of the product between tubes in the first step of preparation [44–46]. This risk can be greatly reduced if transfers are conducted in a laminar flow hood under sterile conditions [7]. In the present study, a one-step PRP preparation protocol using a disposable 6 ml syringe was successfully developed. The one-step syringe centrifugation eliminates the multi-tube transfer process; therefore, the risk of bacterial contamination is greatly reduced. It is important that the venipuncture site be thoroughly cleaned before collecting blood with a sterile needle and syringe. That way, a direct connection is made between the bloodstream of the patient and the sterile equipment. After centrifugation, the blood components can be squeezed into another syringe using a sterile three-way connector without affecting sterility, because the primary blood tube is never opened and the entire process occurs in a closed system [47]. If the syringe is accidentally exposed to the environment, it is considered to be an open system and the product should be discarded to avoid bacterial contaminations. One limitation of the present study was the lack of a bacteria culture. Thus, further studies should assess the effectiveness of the one-step PRP preparation method using syringe centrifugation in reducing bacterial contamination.

Numerous studies have demonstrated that PRP releases several growth factors, especially platelet-derived growth factors. In recent years, the use of PRP in the treatment of degenerative osteoarthritis has increased. Several clinical studies have found that PRP injections have improved functional outcomes and reduced symptoms when compared with hyaluronic acid and placebo controls [13]. Intra-articular injection of PRP also prevented cytotoxic effect of lidocaine on chondrocytes [48] Thus, intra-articular injections of PRP can be useful in angiogenesis and extracellular matrix remodeling, cell proliferation, and differentiation for osteoarthritis [12, 13, 15, 16]. Applications of PRP in fracture repair had been shown in both dogs [49] and rabbit [48] by promoting bone healing. Interestingly, injection of PRP also promoted wound healing of soft tissue defects [50, 51] as well as periodontal regeneration [52]. Since the present study did not evaluate the alteration of growth factors in PRP, it remains unknown whether the use of carprofen as a separation layer affecting the function of platelet-derived growth factors in PRP products. Moreover, several studies including platelet function, markers of platelet storage lesion, and microbial contamination should be evaluated for the safety of the patients [53] should be conducted before clinical application of the single step PRP product.

## Conclusions

The present study revealed that a one-step PRP preparation with carprofen significantly increased platelet yield. The syringe centrifugation method does not require expensive equipment or high technical abilities. Thus, this method can be easily adopted in a veterinary clinic setting. The clinical usefulness of the PRP products derived from the methods used in this study should be investigated further.

## Methods

### Animals

All procedures were approved by the Kasetsart University Institutional Animal Care and Use Committee under approval number #ACKU61-VET-031. A total of 29 healthy dogs (13 males and 16 females) were enrolled in the present study. The dogs weighed 25.0 ± 6.1 kg (mean ± SD) and were 3.5 ± 1.4 years old. Among these 29 dogs were an Alaskan Malamute [1], American Bully [1], Boxer [1], crossbreed [6], Golden Retrievers [7], Labrador Retrievers [3], a St. Bernard [1], and Siberian Huskies [9]. All dog owners signed an informed consent document before the study was conducted. History taking and physical examination were performed by a licensed veterinarian. Complete blood count (CBC), serum biochemistry profiles and quality of blood smear were evaluated before canine blood donation at Kasetsart Pet Blood Bank. Twenty nine bags of blood from these dogs, 14 were used for identifying the optimum force for the one-step PRP preparation with syringe, 12 were used in the one-step syringe centrifugation technique with carprofen, and 3 were used for analysis of platelet morphology.

### Blood collection

None of the dogs were on any medication, including aspirin or other nonsteroidal anti-inflammatory drugs, for 10 days before the experiment. Each dog was given 0.1 mg/kg of xylazine hydrochloride (X-LAZINE®, LBS laboratory, Bangkok, Thailand) intravenously. Approximately 315 ml of whole blood was collected by jugular venipuncture with a 20-gauge needle and deposited directly into 350 ml blood bags containing 35 ml of CPDA-1 (Terumo cooperation, Tokyo, Japan), with a ratio of CPDA-1 to whole blood of 1:9 [7].

### Determination of optimum force for the one-step PRP preparation with syringe

Approximately, 80 ml of donor blood was obtained from each blood bag and 5 ml of blood was drawn into seven 6-ml syringes using an aseptic technique. The present study had modified a syringe method for PRP preparation from previous study [54] and the centrifugation speed and time were adapted from previous studies [7, 54]. To identify the optimal centrifugation force for the one-step PRP preparation, samples were centrifuged at 300, 500, 700, 900, 1100, 1300, and 1500 xg for 5 min at 20 °C. The centrifuge was allowed to stop spinning without operator intervention. After centrifugation, whole plasma fraction containing plasma and platelets was transferred to 3-ml syringe via a sterile three-way. An automated hematology analyzer (Abbott Cell-Dyn 3700, Abbott Park, Illinois, USA) was used to evaluate the number of platelets, red blood cells, and white blood cells. Plasma volume also was measured using laboratory analytical digital balance scales (FX-5000I, Reno, Bangkok, Thailand).

### Determination of optimum force for the one-step PRP preparation with carprofen

Twelve healthy dogs (4 males and 8 females) aged 1–7 years with a mean (± SD) body weight of 26.58 (± 7.35) kg were used to determine the optimum centrifugal force with carprofen (Table 1). Among these 12 dogs were an American Bulldog [1], a Boxer [1], a crossbreed [1], Golden Retrievers [4], a Labrador Retriever [1], and Siberian Huskies [4]. Blood containing CPDA-1 (5 ml) was transferred into four 6 ml syringes. Carprofen (200 μl; Rimadyl®, Norbrook Laboratories Limited Newry, Northern Ireland, UK) was added to act as a separation layer. The blood samples were divided into four aliquots, two containing 5 ml of whole blood without carprofen and two containing 5 ml of whole blood with 0.2 ml of carprofen. To compare the effectiveness of the one-step PRP preparation with and without carprofen, blood samples were centrifuged at four different centrifugation forces (300, 500, 700, and 900 xg), with two replications for each comparison, for 5 min at 20 °C. After centrifugation, the samples were treated in the same fashion as the one-step PRP preparation without carprofen described above. The overall time from blood collection until the retrieval of PRP was under 4 h, hence, the present method is in accordance with the international guideline for safety transfusion [55, 56].

### Scanning electron microscopy (SEM) observation

Three healthy dogs were used to evaluate the morphology of platelets. Five milliliters of blood samples containing CPDA-1 with and without 0.2 ml of carprofen were centrifuged at 500 xg for 5 min at 20 °C. Twenty-five microliters of PRP was dropped on a cover glass and allowed to dry. Blood samples then were fixed using 1.5% glutaraldehyde (Sigma-Aldrich, Missouri, USA) in 0.1 M phosphate buffer, pH 7.2 (Merk, Frankfurter, Germany), at 4^ο^C for 24 h. The specimens were dehydrated through a graded ethanol series, mounted onto aluminum stubs, sputter-coated with platinum, and viewed with a scanning electron microscope (Hitachi SU8020, Tokyo, Japan).

### Statistical methods

The G*Power3.1 (Faul, Erdfelder, Lang and Buchner, 2007) was used to estimate the required sample size using *t*-tests for a paired samples with a power of 90%, and an alpha error of 5% to detect a difference of 1.0 standard deviation of the average value of platelet count. The data were analyzed using R version 3.6.1 (R core team, Vienna, Austria). A Shapiro-Wilk test was used to determine normality. A one-way analysis of variance (ANOVA) was used to compare the different centrifugation forces for the one-step PRP preparation with and without carprofen, as appropriate. The optimal forces were those that gave the maximum values of platelet concentration and plasma. The percentage of platelet recovery in the product of PRP was calculated by multiplying the platelet concentration of the plasma by the plasma volume and then dividing this by the product of the platelet concentration of whole blood and the plasma volume of whole blood and multiplying by 100.

The CV was the measure of relative variability of PRP products for each centrifugation force in the one-step PRP preparation with carprofen. The % CV was calculated by dividing the standard deviation by the mean and multiplying by 100 [26]. Statistical significance was set as *p* < 0.05.

## Data Availability

The data used and/or analyzed in the present study are available from the corresponding author on reasonable request.

## Abbreviations

CV: Coefficient of variation
ml: milliliter
μl: microliter
mg: milligram
PRP: Platelet-rich plasma
RBC: Red blood cell
SD: Standard deviation
SEM: Scanning electron microscopy
WBC: White blood cell
^ο^C: Degrees Celsius
xg: relative centrifugal force
